# Comment on “Olmsted Syndrome”

**DOI:** 10.1155/2020/8024981

**Published:** 2020-06-03

**Authors:** Solene Gatault, Brian Kirby, Dina Danso-Abeam

**Affiliations:** ^1^Charles Institute of Dermatology, School of Medicine and St. Vincent's University Hospital, University College Dublin, Dublin, Ireland; ^2^Systems Biology Ireland, University College Dublin, Dublin, Ireland; ^3^Centre for Colorectal Disease, St. Vincent's University Hospital and School of Medicine, University College Dublin, Dublin, Ireland

We read this paper by Elise Tonoli et al. with interest, and we refer to its Discussion section where three modes of inheritance were proposed for Olmsted syndrome [1]. These were autosomal dominant, X-linked dominant, and X-linked recessive. The X-linked mode of inheritance has been questioned due to recent advancements in and ease of sequencing. We highlight the discrepancies and discuss the literature to date on this issue as follows.

Olmsted syndrome (OS, OMIM# 614594) is a rare hereditary disorder classically marked by bilateral mutilating palmoplantar keratoderma (PPK) and periorificial keratotic plaques [2, 3]. OS has an early onset in the neonatal period or during childhood, and about 73 cases had been reported by the end of 2014 [2]. Subsequently, 33 more new cases have been reported based on PubMed searches: 3 reports with 9 cases in 2015, 6 reports with 14 cases in 2016, 2 reports with 3 cases in 2017, and 5 reports with 7 cases in 2018. No new cases have been reported as of June 2019. Thus, the current total cases of OS reported at the end of June 2019 stands at 106.

The diagnosis of OS is based on the clinical presentation, but there is high variability in the phenotypic spectrum of OS. The classical characteristics are PPK and periorificial keratotic plaques, but it can be accompanied by other features. These include growth retardation, dental anomalies, hearing loss, corneal opacities, leukokeratosis of the tongue or the oral mucosa, diffuse alopecia, nail dystrophy, hyper- or hypohidrosis of the palms and soles, hyperkeratotic linear streaks on the elbows, knees, axillae, and antecubital fossae, and constriction of digits [1, 4]. Altogether, the clinical presentation of OS overlaps with multiple diseases and diagnosis require exclusion of other disorders as outlined by Duchatelet et al. [2].

The mode of inheritance of OS has been controversial. Prior to the identification of the transient receptor potential cation channel, subfamily V, member 3 (TRPV3) as the causative gene in which a mutation leads to OS, three modes of inheritance were proposed based on reported cases [1]. These were autosomal-dominant (OMIM # 614594), X-linked-recessive, and X-linked-dominant (OMIM # 300918) [1]. As more cases were reported post-TRPV3 identification, the proposed modes of inheritance were expanded to include autosomal-recessive by homozygous and compound heterozygous mutations as well as semidominant inheritance [5–7]. So far, the autosomal modes of inheritance have not raised debates in contrast to the X-linked inheritance.

OS patients with X-linked mode of inheritance do not have mutations in TRPV3, but harbour mutations in the membrane-bound transcription factor protease, site 2 (MBTPS2) gene. The controversy is that mutations in MBTPS2 result in X-linked genodermatoses disorders with a variable phenotypic spectrum [8]. Among the disorders, IFAP (ichthyosis follicularis, atrichia, and photophobia) syndrome with or without BRESHECK syndrome (OMIM# 308205) shares an extensive overlapping phenotype with the X-linked form of OS. IFAP syndrome is also a rare disease, and to date, only about 60 cases have been reported worldwide [9]. Ichthyosis follicularis is the most common cutaneous manifestation of IFAP, presenting as noninflammatory follicular keratotic papules, which mainly involve the scalp and extensor extremities. Other cutaneous findings include hyperkeratotic psoriasiform plaques, lamellar scaling, angular cheilitis, periungual inflammation, and dystrophic nails. Nevertheless, unlike OS, there is usually no cornification in the palms or soles in IFAP [3]. Immunological effects have been reported for both OS and IFAP [10, 11].

The phenotypic heterogeneity and overlap of both OS and IFAP make it challenging in arriving at a consensus regarding diagnosis when atypical OS/IFAP cases are presented. For example, clinical features of both IFAP and OS were found in patients in whom a mutation in the MBTPS2 gene has been identified [8]. The phenotype in the patient was designated as “IFAP with Olmsted syndrome-like features” [8]. Thus, Wang et al. raised the following question—does X-linked Olmsted syndrome represent an independent condition or is it merely a severe form of IFAP? [8] This reflection can be discussed by considering the questions asked in the report of Duchatelet et al. First, are there obvious clinical differences between OS caused by mutations in *TRPV3* and *MBTPS2?* Secondly, by what mechanism does mutations in *MBTPS2* lead to IFAP or OS [2]? Reported cases showed that OS caused by mutations in *MBTPS2* usually presents with some IFAP symptoms, while OS caused by mutations in TRPV3 tends to have a classical OS phenotype without IFAP features. Nemer et al. identified a recurrent mutation in *MBTPS2* (p.F475S) in two brothers who were showing clinical features of IFAP and Olmsted syndromes. Interestingly, they showed that WT *MBTPS2* regulates TRPV3 by inducing its activation. This effect was completely lost with the mutant *MBTPS2*. These results suggest an inter-relation between *MBTPS2* and TRPV3 and might explain the overlapping phenotypes frequently observed between the two conditions [12].

The discussion above suggests that “nonclassical” X-linked Olmsted syndrome could be an IFAP syndrome, in which the effect of the mutation in *MBTPS2* interferes with the normal functioning of TRPV3. However, this hypothesis has to be confirmed with a larger sample size population considering genetic, molecular, and functional studies.
